# Spatial dependency of *V. cholera *prevalence on open space refuse dumps in Kumasi, Ghana: a spatial statistical modelling

**DOI:** 10.1186/1476-072X-7-62

**Published:** 2008-12-16

**Authors:** Frank B Osei, Alfred A Duker

**Affiliations:** 1 Department of Geomatic Engineering, Kwame Nkrumah University of Science and Technology (KNUST), Kumasi, Ghana

## Abstract

**Background:**

Cholera has persisted in Ghana since its introduction in the early 70's. From 1999 to 2005, the Ghana Ministry of Health officially reported a total of 26,924 cases and 620 deaths to the WHO. Etiological studies suggest that the natural habitat of *V. cholera *is the aquatic environment. Its ability to survive within and outside the aquatic environment makes cholera a complex health problem to manage. Once the disease is introduced in a population, several environmental factors may lead to prolonged transmission and secondary cases. An important environmental factor that predisposes individuals to cholera infection is sanitation. In this study, we exploit the importance of two main spatial measures of sanitation in cholera transmission in an urban city, Kumasi. These are proximity and density of refuse dumps within a community.

**Results:**

A spatial statistical modelling carried out to determine the spatial dependency of cholera prevalence on refuse dumps show that, there is a direct spatial relationship between cholera prevalence and density of refuse dumps, and an inverse spatial relationship between cholera prevalence and distance to refuse dumps. A spatial scan statistics also identified four significant spatial clusters of cholera; a primary cluster with greater than expected cholera prevalence, and three secondary clusters with lower than expected cholera prevalence. A GIS based buffer analysis also showed that the minimum distance within which refuse dumps should not be sited within community centres is 500 m.

**Conclusion:**

The results suggest that proximity and density of open space refuse dumps play a contributory role in cholera infection in Kumasi.

## Background

The Ganges Delta region is believed to be the traditional home of cholera [1]. From this region, cholera has spread throughout the world, causing seven major pandemics since 1817 [2]. The seventh pandemic, which began in 1961 in Indonesia, reached West Africa in 1970 [3-7]. In Ghana the first bacteriological case report of cholera was on 1^st ^September, 1970 [8]. Since then cholera has been endemic in Ghana, with occasional outbreaks. From 1999 to 2005, the Ghana Ministry of Health officially reported a total of 26,924 cases and 620 deaths to the World Health Organization (WHO) [9-15].

Cholera is an acute intestinal infection caused by the bacterial Vibrio cholerae (*V. cholerae*). The main mode of infection is through contaminated food and drinking water. When ingested in the body, *V. cholerae *produces an exotoxin that either stimulates the mucosal cells to secrete large quantities of isotonic fluid, or increases the permeability of the vascular endothelium, thus allowing isotonic fluid to pass through in abnormal amount [16], resulting in watery diarrhoea. Without prompt treatment *V. cholerae *can cause severe dehydration and death within hours of onset in a severely purging individual [17]. In an unprepared community, case-fatality rate or death can be as high as 50% of severe cases [18,19]. In both concept and execution, *V. cholerae *infection has extraordinarily simple and successful treatment [20]. Oral rehydration salt (ORS) solutions are the most important components of treatment, although intravenous fluids are needed for patients with very severe dehydration [21].

The general assumption by most workers, until quite recently (mid 1960's), was that *V. cholerae *was an organism whose normal habitat was the human gut and/or intestine, and incapable of surviving for more than a few days outside the gut [22]. However, recent studies suggest that the natural habitat of *V. cholerae *is the aquatic environment [23-28]. The ability of *V. cholerae *to survive within and outside the aquatic environment makes cholera difficult to eradicate. Etiological studies suggest that *V. cholerae *survives well in faecal specimens if kept moist [18,29]. When excreted from human faeces and vomitus, *V. cholerae *can survive for up to some days [29]. This makes cholera a complex health problem to manage. Once the disease is introduced in a population, several social, ecological and/or environmental factors may lead to prolonged transmission and secondary cases. Huq et al [30] have specifically demonstrated linkages between cholera and environmental variables. Ali et al [31,32] identified proximity to surface water, high population density, and low educational status as the important predictors of cholera in an endemic area of Bangladesh. Borroto and Martinez-Piedra [33] identified poverty, low urbanization, and proximity to coastal areas as the important geographic predictors of cholera in Mexico. Several scientific studies have also demonstrated the involvement of climatic factors in the recurrence of epidemic cholera [34-36]. However, one very important environmental factor that predisposes inhabitants to cholera infection is sanitation. Since cholera is hypothesized as a disease of deficient sanitation [19], an outbreak of cholera is therefore a stark reminder of deficiency in sanitation systems. Sanitation as a spatial risk can have several measures depending on the geographic area. In Bangladesh, Ali et al [32] used latrine types, classifying them as safe and unsafe, as a measure of sanitation. In studying the geographical patterns of cholera in Mexico, Borroto and Martinez-Piedra [33] also used percentage dwellings without connection to sewerage or septic tanks to incorporate sanitation into a composite poverty index.

In this study, we exploit two main spatial measures of sanitation in an urban city, Kumasi, in a developing country. These are proximity to refuse dumps, and density of refuse dumps within a community. In such settings, significant amount of human excreta reaches refuse dumps. During outbreak periods, surface runoff containing *V. cholerae *contaminate surface water and if consumed perpetuates transmission of the organism. Also, refuse dumps can serve as breeding sites for some dangerous flies. For example, the common housefly, *Musca domestica*, is a eusyanthropic fly species i.e., linked to the human habitat, and a chief offender among the filth breeding flies worldwide [37]. Studies show that the common housefly and flies in general can serve as mechanical vectors of many kinds of pathogens such as bacteria [38,39], protozoa [40], viruses [41], and helminth eggs [42,43]. Our hypothesis is that refuse dumps create environmental niches for *V. cholerae *infection during the rainy season, and therefore inhabitants who live in close proximity to open space refuse dumps should have higher cholera prevalence than those farther. Also areas with high density of refuse dumps should have higher cholera prevalence than areas with lower density. Although cholera is one of the most researched communicable diseases, no study so far has explored the spatial extent at which these environmental factors influence its transmission.

Epidemiologists have long used maps to track the spread of disease, and in the past decade, geographic information system (GIS) technology has added powerful new tools that help reveal far more than simply the "where" and "when" of epidemics [44]. Recent advances in GIS have allowed the application of not only disease mapping but also spatial analysis, such as spatial clustering and cluster detection in epidemiological research [45-47]. A GIS is capable of analyzing and integrating large quantities of geographically distributed data as well as linking geographic data to non-geographic data to generate information useful in further scientific research and in decision making [48]. Spatial analyses (in the context of GIS), such as cluster analysis and geographic correlation studies are commonly used to characterize spatial patterns of diseases [49-55]. Such methodologies have also been utilised in several cholera studies [31-33,56-59]. In this study we use a GIS based statistical modelling to explore the relationship between our spatial measures of sanitation (described above) and cholera prevalence, and spatial scan statistic (SSS) to investigate geographical clusters of cholera. The objectives of this study are (1) determine whether cholera prevalence is related to proximity and density of refuse dumps in Kumasi, (2) detect and map spatial clusters of cholera, and determine whether refuse dumps are a contributory factor to high rate cholera clusters and, (3) to determine a critical buffer distance within which refuse dumps should not be sited away from communities. The results of this study will provide invaluably information to health officials and policy makers to develop effective prevention and control programmes for cholera.

## Results and analysis

### Association between cholera and open space refuse dump

**Figure 1:**
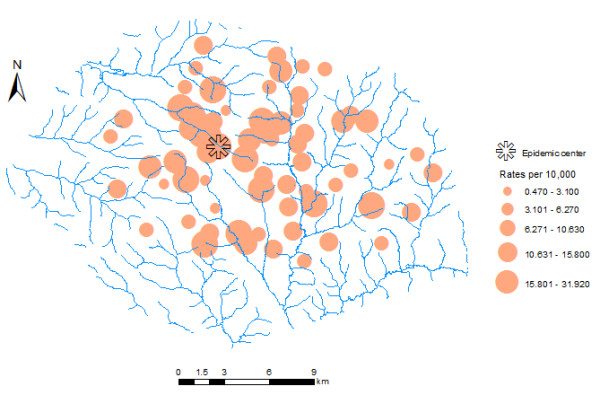
A proportional symbol map showing cholera prevalence for each community.

The summary statistics of the study variables are shown in Table 1. For the period understudy, cholera incidence rates ranged from 0.47 to 31.92 per 10,000 people (mean = 10.21, standard deviation = 6.84). High incidence rates seem to have occurred at the central part of Kumasi (See Figure 1). The results of the spatial regression models are shown in Table 2. Both spatial covariates i.e. nearest distance and dumps density were significantly correlated with cholera incidence. Although both spatial lag and error models are a significant improvement of the OLS model, the spatial error model best fits both covariates (see Table 2). As was expected, a direct spatial relationship between cholera prevalence and *dumps density*, and an inverse relationship with *nearest distance *was observed.

**Table 1:** Summary statistics of variables used for the spatial modelling

Variable	Minimum	Mean	Maximum	Stan Dev
Incidence rate per 10,000 people	0.47	10.21	31.92	6.84

Nearest Distance to refuse dumps (m)	28.75	538.7	2375.5	446.22

Kernel density of dumps (dumps per km^2^)	0	0.29	4.14	0.59

**Table 2:** Results of the spatial regression models; *P*-values are shown in brackets

	***Nearest distance to refuse dumps***			***Kernel density of refuse dumps***		
**Estimation**	**OLS**	**Spatial Lag**	**Spatial Error**	**OLS**	**Spatial Lag**	**Spatial Error**

	12.500	16.911	12.503	8.127	12.63	8.242
Constant	(*0.0001*)	(*0.0001*)	(*0.0001*)	(*0.0001*)	(*0.0001*)	(*0.0001*)

	0.08	0.156	0.175	0.07	0.161	0.174
*R*^2^	(*0.02*)	*	*	(*0.03*)	*	*

	-0.00129	-0.0013	-0.0013	1.858	2.031	1.806
*β*	(*0.02*)	(*0.01*)	(*0.006*)	(*0.03*)	(*0.011*)	(*0.007*)

*δ*^2 ^(Sigma-square)	44.57	39.500	38.640	45.00	39.317	38.688

			-0.468			-0.481
*λ *(lambda)	*	*	(*0.05*)	*	*	(*0.043*)

		0.409			-0.443	
*ρ *(rho)	*	(*0.08*)	*	*	*(0.057)*	*

Log Likelihood	-224.57	-222.311	221.780	-224.906	-222.286	-221.888

Akaike Inf. Criterion	453.15	450.622	447.561	453.812	450.572	447.776

Schwarz Criterion	457.59	457.28	452.000	458.252	457.231	452.215

		4.526	5.58604		5.24	6.036
Likelihood Ratio test	*	(*0.034*)	(*0.02*)	*	(*0.022*)	(*0.014*)

		3.581			4.00	
LM lag	*	(*0.06*)	*	*	(*0.045*)	*

		0.136			0.127	
Robust LM lag	*	(*0.71*)	*	*	(*0.721*)	*

			4.049			4.543
LM error	*	*	(*0.04*)	*	*	(*0.033*)

			0.604			0.669
Robust LM error	*	*	(*0.44*)	*	*	(*0.413*)

	2.24	1.399	1.438	0.992	1.948	2.034
Breusch-Pagan test	(*0.134*)	(*0.24*)	(*0.23*)	(*0.319*)	(*0.163*)	(*0.154*)

* Not available

### Cholera incidence clusters

**Figure 2:**
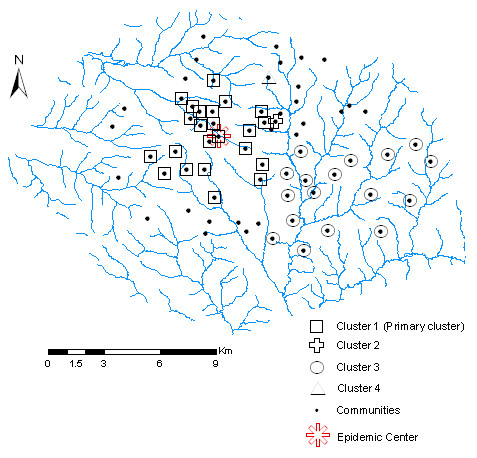
Results of spatial cluster analysis. This map shows the primary cluster with greater than expected cholera prevalence, and two secondary clusters with lower than expected cholera prevalence.

High and low rate spatial clusters of cholera were detected within different window sizes using SSS employed in SaTScan software. Four statistically significant (*P *< 0.001) spatial clusters were detected when the maximum cluster size was = 50% of the total population. The primary cluster (most likely cluster), which was the largest cluster, had greater than expected cholera prevalence rate. This cluster encompassed 23 communities in the study region, where about 27% of the people reside. The overall relative risk (RR) was 1.790, with 376 observed cases compared with about 255 expected cases. This cluster was in areas surrounding the central part of the study region. Of the three secondary clusters with lower than expected prevalence rates, two encompassed a community each, whiles the third encompassed 17 communities where about 30% of the people reside. This cluster surrounded communities located at the south eastern part of the study region (Figure 2 and Table 3).

**Table 3:** Results of cholera clusters using spatial scan statistics

**Cluster**	**No. Communities**	**No. of cases**	**Exp. No. cases**	**Population**	**RR**	***P*-value**
1	23	376	254.52	268295	1.790	0.001

2	1	1	20.19	21281	0.049	0.001

3	17	214	279.97	295113	0.696	0.001

4	1	23	53.52	56417	0.416	0.001

When spatial models were built within the cluster with higher than expected cholera prevalence, a result similar to the model within the whole study region was obtained. However, neither the spatial lag nor spatial error variables were included in this model due to the absence of spatial dependency in the residuals. Hence OLS model best fitted the cluster with higher than expected prevalence. Cholera prevalence was positively associated with density of refuse dumps (*R*^2 ^= 0.10, *P *< 0.1), and negatively associated with proximity to refuse dumps (*R*^2 ^= -0.24, *P *< 0.01). However, association between cholera prevalence and density of refuse dumps was only significant at 10%.

### Critical buffer distance

**Figure 3:**
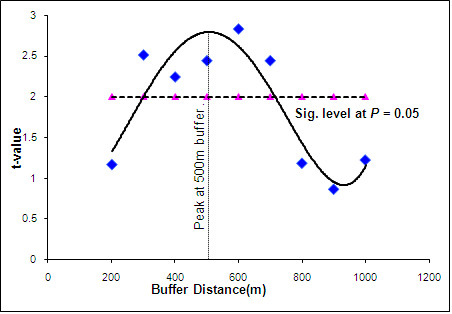
Quantitative assessment of critical distance discrimination obtained by applying experimental buffer zones around refuse dumps.

The result of the *t*-tests statistics for significant spatial determination of critical buffer distance is shown in Table 4 and Figure 3. For the buffers tested, *t values *ranged from 0.88 to 8.86 whereas mean cholera prevalence ranged from 4.5 to 21.67 per 10,000. A quantitative assessment of distance discrimination of the experimental buffer zones around refuse dumps shows that the optimum spatial discrimination of cholera occurs at 500 m from refuse dumps (Figure 3). With this buffer 42 of the 68 communities (i.e., 62%) fall within the 500 m distance to refuse dumps. For communities within this buffer, cholera prevalence ranged from 3.93 to 31.92 cases per 10,000 people, and mean prevalence of 11.77. For communities beyond this buffer, cholera prevalence ranged from 1.3 to 21.73 cases per 10,000 people, and mean prevalence of 7.71 (Table 4).

**Table 4:** Results of buffer distances and associated *P*-values

**Buffer distance(m)**	**200**	**300**	**400**	**500**	**600**	**700**	**800**	**900**	**1000**
Mean within buffer	12.56	12.77	11.9	11.77	11.82	11.49	10.72	10.54	10.63

Mean outside buffer	9.81	8.48	7.95	7.71	7.07	7.17	8.28	8.67	7.77

*t*-statistic	1.164	2.51	2.42	2.44	2.83	2.44	1.18	0.86	1.22

*P*-value	0.12	0.007	0.009	0.007	0.003	0.008	0.12	0.20	0.114

## Conclusion

The results of this study reveal the spatial dependency of cholera infection upon proximity and density of refuse dumps in Kumasi. This means that refuse dumps serve as niches for cholera infection. The results also show that the minimum distance within which refuse dumps should not be located from a community is 500 m. We therefore hypothesize that proximity and density of refuse dumps may play a significant role in cholera transmission. House-to-house collection of refuse, which is at a limited service, should therefore be extended to all communities within the Kumasi metropolis.

This study has investigated only the influence of refuse dumps on cholera prevalence. Further rigorous research is needed to determine whether other demographic and environmental factors are involved. Moreover, some of the findings of this study, i.e. possible involvement of flies in cholera transmission, are only indicative, and require a confirmation by environmental and/or epidemiological research.

## Materials and methods

### The study area

**Figure 4:**
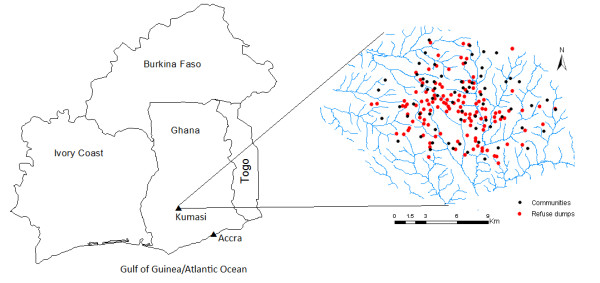
District Map of Ghana (left), and Kumasi (right).

This study was conducted in Kumasi, the capital of Ashanti Region (See Figure 4). Ashanti Region is centrally located in the middle belt of Ghana. It lies between longitudes 0.15°W and 2.25°W, and latitudes 5.50°N and 7.46°N. The region is divided into 18 administrative districts. The Kumasi metropolis alone accounts for nearly one-third of the region's population [60]. Kumasi is located in the south-central part of the country, about 250 km (by road) northwest of Accra, the capital city of Ghana (Figure 4). It lies at the intersection of latitude 6.04°N and longitude 1.28°W, covering an area of about 220 square kilometers. The Kumasi metropolis is the most populous district in the region. It has a population of about 1.2 million which accounts for just under a third (32.4%) of the region's population. Kumasi has attracted such a large population partly because it is the regional capital, and also the most commercialized town in the region. Other reasons include its centrality as a nodal town with major road arteries to other parts of the country. A greater proportion of households in the metropolis, 81.2%, use the public dump to dispose of solid waste. Only 2.2% of the metropolis population's wastes are collected (i.e. house to house collection), but only in few first class residential areas. The remaining population either burry their waste, burn or dump it elsewhere [60]. Most of these refuse dumps also serve as transfer stations for transferring waste to a landfill site. Sanitation generally becomes a problem during the rainy season. Most access roads to refuse dumps are unpaved and become extremely deplorable during the rainy season, and therefore refuse collection vehicles are not able to ply such roads. As a result, refuse are left to pile up and spread during the rainy season. The main source of drinking water in the metropolis is pipe borne water (about 82%). Nearly 11.5% drink from well, 1.5% drink from river, pond or lake, while 0.8% of the people obtain their drinking water from tanker supply [60]. However, due to rampant water shortages, most inhabitants resort to nearby streams and rivers for various household activities such as cooking and washing.

### Cholera case definition and data

In Ghana, a suspected case of cholera is based on the WHO's definition which depends on whether or not the presence of cholera has been demonstrated in the area. According to the WHO [19] guidance on formulation of national policy on the control of cholera, in an area where the disease is not known to be present a case of cholera should be suspected, when a patient, 5 years of age or older develops severe dehydration or dies from acute watery diarrhoea, or where an epidemic is occurring, a patient, 5 years of age or older develops acute watery diarrhoea, with or without vomiting. However, the first suspected case of cholera has to be confirmed by bacteriological tests (personal communication with Kumasi Metro Health Directorate (KMHD) director).

During the year 2005 severe outbreaks of cholera occurred in most urban communities in Ghana. In Kumasi, this outbreak started from the last week of September, lasting for a period of 72 days, which was within the rainy season. The first suspected and confirmed case was recorded on 29^th ^September, 2005. The outbreak source was traced to a slum settlement (Racecourse) which is an abandoned racecourse. All cholera data for this study were obtained from the Kumasi Metropolitan Disease Control Unit (DCU), since it is mandatory for all reporting facilities (i.e. hospitals, clinics, and community volunteers) to report weekly cholera cases to the DCU. According to the DCU, cholera surveillance and reporting before 2005 has been ineffective, and hence the existing data before 2005 has little or no spatial information. However, with intensified surveillance and reporting systems during the 2005 outbreak, cases were recorded at community level (spatial unit of reporting). Therefore, this study utilised only cholera cases reported during the 2005 outbreak.

### Refuse dumps data

A Global Positioning System (GPS) was used to determine the geographic coordinates of all refuse dumps. The geographic coordinates (latitudes and longitudes) in the WGS 84 datum were then transformed into the Ghana Transverse Mercator (GTM) coordinate system using a simple transformation program written by one of the authors (FBO) in Microsoft Visual Basic for application (VBA) for Microsoft Office Excel 2003. The GTM coordinates were then imported into a GIS for mapping and further analysis. A total of 124 refuse dumps were mapped. Based on the hypothesis that cholera is a disease of deficient sanitation, the following predictions were made: inhabitants who live in close proximity to open space refuse dumps should have higher cholera prevalence than those farther. Also areas with high density of open space refuse dumps should have higher cholera prevalence than areas with lower density.

### Spatial data input

Topographic map of the study area at a scale of 1:2500 obtained from the planning unit of the Kumasi Metropolitan Assembly was digitized using ArcGIS version 9.0 developed by Environmental System Research Institute (ESRI). Before digitizing, the map was georeferenced (by defining the X and Y coordinates of corner points of the map) into the GTM coordinates system. The main boundary was digitized as a polygon feature while the locations of communities were digitized as point features. Reported cases of cholera in 2005 obtained from the KMHD were entered as attributes of the point features. Population estimates for 2005, obtained from the Ghana Statistical Service (GSS), were used in calculating the raw rates of cholera. Raw rates were calculated as the number of cholera cases in each community divided by the estimated population in 2005. In order to express the notion of risk more intuitively, the raw rates were rescaled by multiplying it by a factor, i.e. 10,000. This expresses the raw rate as per 10,000 people. The resulting layer was then overlaid on the refuse dumps layer for further analysis.

### Spatial data analysis and statistical modelling

House-to-house collection of waste in Kumasi is limited to only few first class residential areas. The rest of the population exploit open spaces and approved demarcated parcels as refuse dumps. Due to the rate of urbanisation and population growth, most of these refuse dumps have now reached the centres of communities and overcrowded areas. Inhabitants in close proximity to refuse dumps are assumed to have a higher risk of contracting cholera. Also areas with high density of refuse dumps should have higher cholera prevalence than areas with lower density. Spatial analysis was therefore used to determine the spatial relationship between cholera prevalence per community and (a) proximity (distances) to refuse dumps, (b) density of refuse dumps.

**Figure 5:**
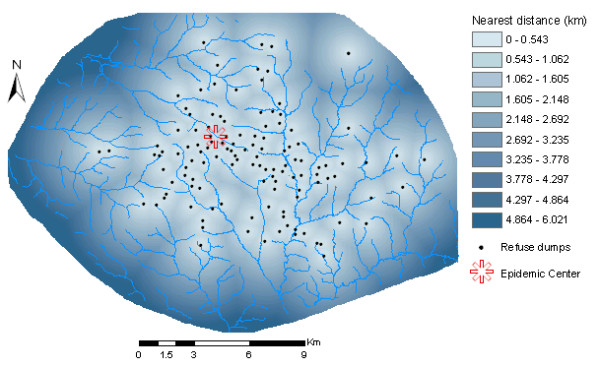
Distance surface (after neighbourhood statistics), showing distances from each pixel to the nearest potential cholera source (refuse dumps).

**Figure 6:**
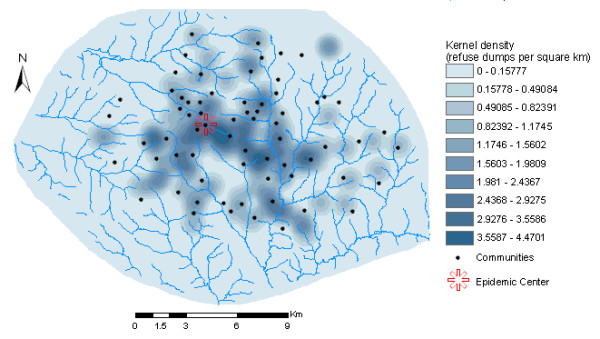
Kernel density surface (after neighbourhood statistics), showing the number of refuse dumps per unit area.

Spatial analysis was carried out in two principal steps. Firstly, two spatial factor maps were generated: (a) *spatial distance surface*, showing distances of each point (cell or pixel) to the nearest refuse dump (Figure 5); (b) *kernel density surface*, showing the number of refuse dumps per unit area (Figure 6). Kernel density calculates the density of point features around each output raster cell. In this concept, smooth curved surfaces are fitted over each point. The surface value is highest at the location of the point and diminishes with increasing distance from the point, reaching zero at a search radius distance from the point. The density at each output raster cell is calculated by adding the values of all the kernel surfaces where they overlay the raster cell centre. The kernel function is based on the quadratic kernel function described in Silverman [61]. In this study, a search radius of 1 km was used to calculate the kernel density.

To finally create the spatial factor maps, spatial neighbourhood statistics was performed on both the distance and density surface maps to calculate the mean pixel values within a neighbourhood of 1 km radius. The spatial factor maps were subsequently crossed with the point map of communities to create two spatial covariates: (a) *Distance*: distances from each community to the nearest refuse dump; (b) *Density*: number of refuse dumps per unit area for each community. Summary statistics of the variables used for modelling are shown in Table 1.

Secondly, using the spatial covariates as explanatory variables, we developed a set of spatial models that attempted to relate cholera incidence rates to refuse dumps in Kumasi. Spatial regression methodologies in the context of spatial econometric framework [62-64] were used for the modelling. When standard linear regression, i.e. Ordinary Least Squares (OLS) models are estimated for cross-sectional data on neighbouring spatial units, the presence of spatial dependence may cause serious problems of model misspecification. The methodologies of spatial regression consist of examining and testing for the potential presence of such misspecifications and providing a more appropriate modelling that incorporates the spatial dependence [65,66].

In matrix notation, the general form of OLS estimation model is given by:

(1) $$y_{i}=\beta {X^{\prime }}_{i}+\varepsilon_{i},$$

where *y*_*i *_is an observation on the dependent variable, ${X^{\prime }}_{i}$ is an observation on the explanatory variable (distance variable or density variable), with *i *= 1 ....., *N *(including a constant term, or 1), and *β *is the matching regression coefficient for the explanatory variable, where *ε*_*i *_is a random error term. In this model, the error terms are assumed to have zero mean (*E *[*ε*_*i*_] = 0, ∀ *i*), and are identically and independently distributed (*i.i.d*). Consequently, their variance is constant, *Var *[*ε*_*i*_] = *σ*^2 ^and they should be uncorrelated, i.e. (*E *[*ε*_*i*_*ε*_*j*_] = 0, ∀ *i, j*). These assumptions are usually violated due to the presence of spatial dependence (spatial autocorrelation) in the residuals of the OLS model and consequently, model misspecification. Spatial dependence can be incorporated into the OLS model in two distinct ways: as an additional predictor in the form of a spatially lagged dependent variable (spatial lag model), or in the error structure (spatial error model).

In a spatial lag model, the spatial lag variable is introduced at the right hand side of equation (1) as:

(2) $$y_{i}=\rho {y^{\prime }}_{i}+\beta {X^{\prime }}_{i}+\varepsilon_{i},$$

*ρ *is an autoregressive coefficient of the lag variable ${y^{\prime }}_{i}$. The spatial lag variable in the model can be expressed as:

${y^{\prime }}_{i}=\sum_{j}w_{ij}y_{i}$, where *w*_*ij *_is raw standardised spatial weight matrix, hence $\sum_{j}w_{ij}=1,\forall i$.

The spatial error variable can also be introduced in the OLS model as:

(3) $$y_{i}=\beta {X^{\prime }}_{i}+\Phi_{i},$$

with $\Phi_{i}=\lambda \sum_{j}w_{ij}\Phi_{i}+\nu_{i}$ where Φ_*i *_is the error vector of the random error term assumed to be *i.i.d*, *λ *is the spatial autoregressive coefficient and *ν *is considered to be a white noise error. The errors Φ_*i *_are assumed to follow a spatial autoregressive process with autoregressive coefficients *λ*.

The parameters of the spatial lag and error models were estimated by means of the maximum likelihood (ML) method (that is, the parameters are estimated by maximizing the probability/likelihood of the sample data). ML estimation of spatial lag and spatial error regression models were first outlined by Ord [67]. The log likelihood function for spatial lag model is given by:

$$\begin{array}{r} \ln L=-(N/2)\ln(2\pi )-(N/2)\ln\sigma^{2}+\ln\left|I-\rho W\right|\\ -(1/2\sigma^{2})(y-\rho Wy-X\beta {)}^{\prime }(y-\rho Wy-X\beta ) \end{array},$$

where I is a N by N identity matrix.

The first conditions for the ML estimators yield nonlinear (in parameters) equations which are solved by numerical methods. For a ML estimate for *ρ *it is obtained from a numerical optimization of the concentrated log-likelihood function.

The maximum likelihood estimation for the spatial error model employs the error covariance term into log-likelihood function as follows:

$$\begin{array}{c} \ln L=-(N/2)\ln(2\pi )-(N/2)\ln\sigma^{2}+\ln\left|I-\lambda W\right|\\ -(1/2\sigma^{2})(y-X\beta {)}^{\prime }(I-\lambda W{)}^{\prime }(I-\lambda W)(y-X\beta ) \end{array},$$

As in spatial lag model, the ML estimate can also be solved numerically and the estimates are obtained from the optimization of a concentrated log-likelihood function.

### Model specification and selection

A widely used diagnostic test for spatial error dependence is an extension of Moran's I to the regression context. In addition, Anselin [68] provides the best guidance for model specification based on the joint use of the Langrage Multiplier (LM) tests for spatial lag and spatial error dependence. When Moran's I statistic for the error terms of the OLS model is significant, LM test for spatial lag and spatial error dependence is used. When both tests have high values indicating significant spatial dependence in the data, the one with the highest value (lowest probability) will indicate the proper specification.

The test statistics for spatial error dependence is constructed from the OLS residuals, and it is given by:

I = *e*'*We/e*'*e*,

where e is an N by 1 vector of regression residuals from the OLS estimation on a sample with N observations, and *W *is N by N weights matrix (typically row-standardized). Inference is based on the normal distribution.

The LM-lag statistic has the following form:

$$LM(Lag)=\frac{{\left(e^{\prime }Wy/s^{2}\right)}^{2}}{RJ_{\rho -\alpha }},$$

where, e is a vector of OLS residuals, y is the dependent variable and *RJ*_*ρ*-*α *_= [*T *+ (*Wβ a*)' *M*(*Wβa*)/*s*^2^], where *T *= *tr*(*W*'*W*+*W*^2^), with *tr *as the matrix trace operator, and *M *= *I *- *β*(*β*'*β*)^-1^*β' *is the projection matrix. The statistic is distributed as *χ*^2 ^with one degree of freedom.

The LM-error test for spatial error dependence was suggested by Burridge [69] and Anselin [68], and has the following form:

$$LM(error)=\frac{\left(e^{\prime }We/s^{2}\right)}{T}.$$

**Figure 7:**
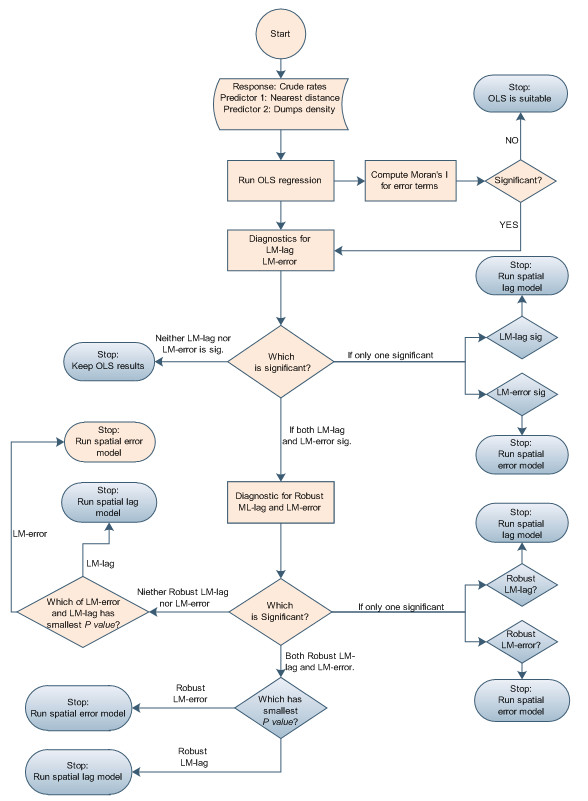
Flow chart showing step by step decisions for the spatial modelling. The shapes in pink show the decisions made to select the appropriate model that best fit our data.

This statistic is also distributed as *χ*^2 ^with one degree of freedom. The best model that fits the data was based on the computed test statistics, and selected using a step by step procedure shown in the flow chart (See Figure 7).

### Spatial clusters detection

One of the most important statistical tools for cluster detection, SSS was used to detect most likely clusters for both high and low rates of cholera incidences. SSS has a disadvantage of being difficult to incorporate prior knowledge about the size and shape of an outbreak and its impact on disease rate [70]. We took advantage of this disadvantage to get rid of pre-selection biases of clusters and their locations.

SSS is based on the likelihood ratio test, and analyses have been available for count data using either a Poisson or Bernoulli model [71]. This method is based on the principle that the number of cholera cases in a geographic area is Poisson-distributed according to a known underlying population at risk [72]. SSS software, SaTScan [73], was used for all cluster analysis. A circular window was imposed on the study region which is moved over the region and centred on the centroid of each community. The size of the circular window or the cluster size was expressed as a percentage of the total population at risk. This varied from 0 to a maximum (not exceeding 100), as specified by the user, expressed as a percentage of the population at risk. In this study, the retrospective spatial cluster analysis for both high and low rates was used, that is when SaTScan evaluates all temporal windows less than the specified maximum window size.

The maximum window size never exceeded 50% of the total population because clusters of larger sizes would indicate areas of exceptionally low rates outside the circle rather than areas of exceptionally high rates within the circle. Possible clusters were tested within the window whenever it was centred on the centroid of each community. Whenever the window finds a new case, the software calculates a likelihood function to test for elevated risk within the window in comparison with those outside the window. The likelihood function for any given window was proportional to:

$$\lambda ={\left(\frac{d}{n}\right)}^{d}{\left(\frac{D-d}{D-n}\right)}^{D-d}I()$$

Where *D *is the total number of cases, *d *is the number of cases within the window, *n *is the expected number of cases and *I*() is the indicator function [72].

The indicator function, *I*(), depended on the comparison between the expected number of cases and the reported number of cases. *I*() was 1 when the reported number of cases within the window was more than the expected number of cases, otherwise 0. The test of significance level of clusters was through the Monte Carlo hypothesis testing. This was used to test for the significance of the cluster that is least likely to have occurred by chance. The null hypothesis of no cluster was rejected when the simulated *P value *was less than or equal to 0.05 for most likely clusters and 0.1 for secondary clusters since the latter have conservative *P-values *[72].

To investigate whether proximity and density of open space refuse dumps was associated with cholera within the cluster with higher than expected cholera prevalence (i.e. primary cluster), a similar modelling approach was applied (See *Spatial data analysis and statistical modeling*).

### Critical buffer distance

A major significance of this research is to give recommendation to public health officials and town planners about the critical (minimum) distance within which refuse dumps should not be sited (away from inhabitants of communities). A GIS based buffer analysis and statistical analysis was used to estimate this critical distance. In this analysis, a buffer zone was defined as a specified distance around a selected map feature [48]. Firstly, a series of *Boolean-distance *maps for experimental buffer distances from 200 m to 1000 m at regular buffer intervals of 100 m were created around refuse dumps. Boolean maps were created in such a way that distances less or equal to a buffer distance were considered high risk zones, whereas distances greater than the buffer distance were considered as low risk zone. Within each buffer zone, the mean cholera prevalence rate was computed. At each buffer zone, a test of the significance of the difference of mean cholera prevalence within the buffer and outside the buffer was calculated using the *t*-statistic:

tij=x¯i−x¯jsp2(1/ni+1/n)j,

where ${\bar{x}}_{i}$, ${\bar{x}}_{j}$ are sample means, $s_{p}^{2}$ is the polled sample variance, *n*_*i *_and *n*_*j *_the sample sizes from population *i *and *j*. Using *t*_*ij *_and degrees of freedom given by *n*_*i *_+ *n*_*j *_- 2, a *t *distribution look-up table provides the probability, *P *that the means are significantly different.

## Discussion

### Association between cholera and refuse dumps

The results of our spatial regression models suggest that proximity to and density of refuse dumps are the important environmental predictors of cholera (epidemic) in Kumasi. Cholera has been generally hypothesized as a disease of deficient sanitation. Since proximity and density of refuse dumps can serve as an index of basic sanitation within an area, our findings support the general hypothesis of cholera. However, our measure of sanitation i.e. proximity to and density of refuse dumps makes our study different from other studies. Two main reasons may explain the plausibility of our findings.

#### (1) High rate of contact with filth breeding flies

Flies are attracted by the odour emanating from refuse dumps, especially the common housefly. This fly lives in close association with man feeding on all kinds of human food, garbage and excreta, and will travel no farther from its breeding site (refuse dumps) to the nearest resting place. The indiscriminate feeding habits (feeding on filth and human food) of this fly species combined with its structural morphology (presence of hair and sticky pads) make them ideally suited to carry and disseminate pathogenic micro organisms [37,74,75]. Research has proven that the common housefly (*Musca domestica vicina*) and flies in general are mechanical vectors of many kinds of pathogens such as bacteria [38,39], protozoa [40], viruses [41], and helminth eggs [42,43]. Fotedar [76] undertook a study to ascertain the vector potential of the domestic housefly as a carrier of *V. cholerae *in Delhi, India, where an outbreak of cholera was encountered. Viable *V. cholerae *was isolated from six (60%) of the pooled fly samples, which confirmed that there were potentially contaminated mechanical vectors among the flies. Some published reports have also shown that fly control measures can be effective in reducing the incidence of diarrhoea [39,77,78]. Where high fly populations and poor hygiene conditions prevail, or where pathogens can grow within fly-contaminated food, the potential exists for transmitting pathogens with a high infectious dose (e.g., *V. cholerae*, Salmonella spp.) [79]. Studies also show that *V. cholerae *is able to colonize and multiply within some flies like the *Drosophila melanogaster *[80]. Although *V. Cholerae *infection is dose dependent (about 10^8 ^cells) [81,82], and the number of bacterial cells flies can carry is not clear, flies can contaminate food in which the cholera vibrios could multiply and reach an infective dose. It is worth nothing that this study does not prove fly transmission of *V. Cholerae *to humans, but only gives an indication of their possible involvement in transmission. Therefore, further epidemiological and fly control intervention studies during an outbreak period are required to emphatically prove this hypothesis.

#### (2) Flood water contamination

Significant amount of human excreta ultimately reaches solid waste systems through dump diapers, faeces of children, or even adults faeces are directly added to the solid waste in the homes. Some people also defecate along roadways, streets, and areas which are swept by public sweepers. These fecal matters also end up in the solid waste. Most often, the excreta of young children are also considered to be harmless, and hence end up in solid waste systems. Etiological studies have shown that *V. cholerae *survives well in faecal specimens if kept moist [18]. When excreted from human faeces and vomitus, *V. cholerae *can survive up to some days whiles moist [29]. In the period of cholera outbreak, runoff from open space dumps during heavy rains may serve as the major pathway for the distribution of the bacteria, creating environmental niches for the bacteria infection. Excreta may be washed away by rain-water and can run into nearby wells, streams and surface water bodies. The bacteria in the excreta may then contaminate these water bodies. The Kumasi metropolis suffers from frequent sporadic water shortages. During such periods, the people exploits nearby streams and surface water bodies for cooking, drinking, bathing and other activities which can perpetuates the transmission of the bacteria.

This finding is similar to the classic epidemiological study of John Snow in the 1850's in London, when he showed the association of cholera with contaminated drinking water even before any bacterial were known to exist [83,84]. In those days, people didn't have running water or modern toilets in their homes. They used town wells and communal pumps to get the water for drinking, cooking and washing. Septic systems were primitive and most homes and businesses dumped untreated sewage and animal waste directly into the Thames River or into open pits called "cesspools". However, water companies often bottled water from the Thames and delivered it to pubs, breweries and other businesses. This caused massive water contamination and led to a severe cholera outbreak in the Soho district of London, causing over 600 deaths. By using a geographical grid to chart deaths from the outbreak and investigating each case, Snow realised that only people who used bottled water from the Thames River were infected. He also realised that sewage dumped into the river or into cesspools near town wells contaminated the water supply, leading to a rapid spread of disease.

### Cholera incidence clusters

Four statistically significant spatial clusters of cholera were identified, one significant most likely cluster with greater than expected prevalence rates, and three secondary clusters with lower than expected prevalence rates. The most likely cluster which was the largest cluster (See Table 3 and Figure 2) encompassed 23 communities, where 27% of the people reside. For this cluster, 21 out of the 23 communities were within the critical buffer distance (i.e. within 500 m of refuse dumps). This indicates that proximity to refuse dumps might be a significant contributory factor to the high rates of cholera. When spatial models were built within this cluster, a result similar to the model within the whole study region was obtained, only that association between cholera prevalence and density of refuse dumps existed only at 10% significance level. Also, this cluster was concentrated within the central part of the study area, around the epidemic centre (Racecourse, Bantama), i.e. where the epidemic began. The reasons for this could be several. Firstly, cholera is a contagious disease which can diffuse from its source of outbreak to other communities based on their proximity; hence high incidence rates are likely to occur at areas proximal to the outbreak source than areas farther. Secondly, the outbreak source, Racecourse, is a waterlogged area and a forcibly created market centre with high commercial activities. This area generally has insufficient public toilets and garbage bins to accommodate the large daily influx of traders. Consequently, traders and buyers may resort to unsanitary practices, urinate and defecate into open gutters and other open spaces and into polythene bags, which ultimately create unsanitary conditions for livelihood. Since cholera outbreak is a stark reminder of deficiency in good sanitation practices, the patterns displayed may be partly explained by the above mentioned reasons. The cluster with lower than expected prevalence rates was concentrated at the south eastern part of Kumasi. Most of the communities within this cluster are residential and peri-urban areas where population density and commercial activities are relatively minimal. House to house collection of refuse has also been successfully achieved at the residential communities, hence dumping of refuse in open spaces is scarcely found.

### Critical buffer distance

A major significance of this research was to give recommendation to public health officials and town planners about the critical (minimum) distance within which dumps should not be sited (away from inhabitants of communities). From the results, it is evident that the critical buffer distance within which refuse dumps should not be sited is 500 m (see Figure 3). This buffer distance included about 62% of communities, where about 68% of the people reside.

The city's expansion both spatially and in population has strained existing resources meant to achieve effective waste management systems (example *house to house *collection of waste). This has led to the creation of many open space dumps very close to community centres. Consequently, about 62% of communities have their refuse dumps within the critical buffer distance (*buffer distance with the lowest P-value*). Since cholera outbreaks are as a results of poor sanitation, opens space refuse dumps within community centres can predispose inhabitants to cholera infection.

This present study provides useful information about the location of clusters of cholera and an environmental factor that might have led to increased cases during the period of the outbreak. This new knowledge, the spatial dependency of high cholera prevalence on refuse dumps location will be useful to health officials and policy makers to make appropriate decisions. With the critical buffer distance identified in this study, it cautions policy makers not to locate any open space refuse dump within 500 m radius from the centre of any community.

This study also has some potential limitations. Firstly, the data used is for only a single year outbreak. The best approach was to use data from several cholera outbreaks; however, cholera reporting at a relatively smaller spatial scale (community level) has not been available before the 2005 outbreak. Secondly, the assumption was that the population within a community has equal risk of exposure to refuse dumps. In reality, this is not so because within a particular community, individuals living close to refuse dumps have a higher risk of exposure than those living farther away. Thirdly, this study could not correlate periods of water shortages with the outbreak period of cholera.

## Competing interests

The authors declare that they have no competing interests.

## Authors' contributions

FBO carried out the research and drafted the manuscript. AAD guided the research and reviewed the manuscript. All authors read and approved the final manuscript.
